# Pygenic Acid A (PA) Sensitizes Metastatic Breast Cancer Cells to Anoikis and Inhibits Metastasis In Vivo

**DOI:** 10.3390/ijms21228444

**Published:** 2020-11-10

**Authors:** Ga-Eun Lim, Jee Young Sung, Suyeun Yu, Younmi Kim, Jaegal Shim, Hyo Jung Kim, Myoung Lae Cho, Jae-Seon Lee, Yong-Nyun Kim

**Affiliations:** 1 Division of Translational Science, National Cancer Center, 323 Ilsan-ro, Ilsandong-gu, Goyang-si, Gyeonggi-do 10408, Korea; 74621@ncc.re.kr (G.-E.L.); sungjy@ncc.re.kr (J.Y.S.); biosuyeun@ncc.re.kr (S.Y.); younme_810@naver.com (Y.K.); jaegal@ncc.re.kr (J.S.); 2 National Institute for Korean Medicine Development, 94 Hwarang-ro (Gapje-dong), Gyeongsan-si, Gyeongsangbuk-do 38540, Korea; indersee31@nikom.or.kr (H.J.K.); meanglae@nikom.or.kr (M.L.C.); 3 Department of Molecular Medicine, College of Medicine, Inha University, 100 Inha-ro, Michuhol-gu, Incheon 22212, Korea; jaeslee@inha.ac.kr

**Keywords:** anoikis, cell aggregations, metastasis, breast cancer

## Abstract

Metastasis is the main cause of cancer-related deaths. Anoikis is a type of apoptosis caused by cell detachment, and cancer cells become anoikis resistant such that they survive during circulation and can successfully metastasize. Therefore, sensitization of cancer cells to anoikis could prevent metastasis. Here, by screening for anoikis sensitizer using natural compounds, we found that pygenic acid A (PA), a natural compound from *Prunella vulgaris*, not only induced apoptosis but also sensitized the metastatic triple-negative breast cancer cell lines, MDA-MB-231 cells (human) and 4T1 cells (mouse), to anoikis. Apoptosis protein array and immunoblotting analysis revealed that PA downregulated the pro-survival proteins, including cIAP1, cIAP2, and survivin, leading to cell death of both attached and suspended cells. Interestingly, PA decreased the levels of proteins associated with anoikis resistance, including p21, cyclin D1, p-STAT3, and HO-1. Ectopic expression of active STAT3 attenuated PA-induced anoikis sensitivity. Although PA activated ER stress and autophagy, as determined by increases in the levels of characteristic markers, such as IRE1α, p-elF2α, LC3B I, and LC3B II, PA treatment resulted in p62 accumulation, which could be due to PA-induced defects in autophagy flux. PA also decreased metastatic characteristics, such as cell invasion, migration, wound closure, and 3D growth. Finally, lung metastasis of luciferase-labeled 4T1 cells decreased following PA treatment in a syngeneic mouse model when compared with the control. These data suggest that PA sensitizes metastatic breast cancer cells to anoikis via multiple pathways, such as inhibition of pro-survival pathways and activation of ER stress and autophagy, leading to the inhibition of metastasis. These findings suggest that sensitization to anoikis by PA could be used as a new therapeutic strategy to control the metastasis of breast cancer.

## 1. Introduction

Pygenic acid A (PA; 3-epicorosolic acid, corosolic acid) is a natural compound that is used in oriental medicine and is extracted from *Prunella vulgaris*, a perennial herb that is widely distributed throughout the world. PA has been demonstrated to have various effects on diseases, such as diabetes [1] inflammatory diseases [2], and cancers [3]. PA has been reported to promote β-catenin degradation through the proteasomal pathway and inhibit colon cancer proliferation [4]. In addition, PA inhibited HER2(ERBB-2) signaling cascade, which is involved in cell cycle progression, in gastric cancer cells [3]. Although there have been several reports on the anti-tumor effects of PA and the anti-metastatic effects of PA, the mechanisms involved in these events have not yet been comprehensively studied.

Tumor metastasis is a multistep process, involving the dissociation of cancer cells from the primary tumor site, intravasation allowing cells to enter blood stream, migration through the circulatory system, and extravasation, resulting in cells settling and growing in a secondary organ [5]. When cancer cells travel through the circulatory system, they encounter hurdles, such as shear stress and anoikis, which they must overcome to successfully metastasize [6]. Anoikis is a form of cell death induced by cell detachment from the extracellular matrix (ECM) and cell-cell interaction. Normal cells are sensitive to anoikis and so undergo apoptosis when they become detached from the ECM. However, cancer cells acquire anoikis-resistance when they become detached. Because of this anoikis resistance, cancer cells can survive in circulation and disseminate to secondary sites to form tumor metastases [7]. Therefore, the sensitization of cancer cells to anoikis is critical for the control of metastasis.

We and others have demonstrated that cell aggregate formation is critical for the development of anoikis resistance during suspension cultures [8,9]. Previously, we reported that BEAS-2B normal human lung epithelial cells form fewer and smaller cell aggregates, and, thus, undergo anoikis when grown under suspension culture conditions. However, A549 human lung cancer cells form lager cell aggregates faster than BEAS-2B. A549 cells are anoikis-resistant and they survive and grow in suspension culture conditions through the NADPH oxidase 4 (NOX4)-EGFR pathway [8]. Apocynin, a NOX4 inhibitor, prevents cell aggregation and thus induces anoikis in suspension cultures [8]. Therefore, the inhibition of cell aggregation in suspension culture, thereby sensitizing anoikis could be used as a strategy to discover potential therapeutic drugs to control metastasis. In this study, we demonstrate that PA effectively sensitizes metastatic breast cancer cells to anoikis via the inhibition of multiple signaling pathways and thus inhibits tumor metastasis in the syngeneic mouse model.

## 2. Results

### 2.1. Pygenic Acid A (PA) Induces Cell Death and Sensitizes Metastatic Breast Cancer Cell Lines to Anoikis

**Figure 1:**
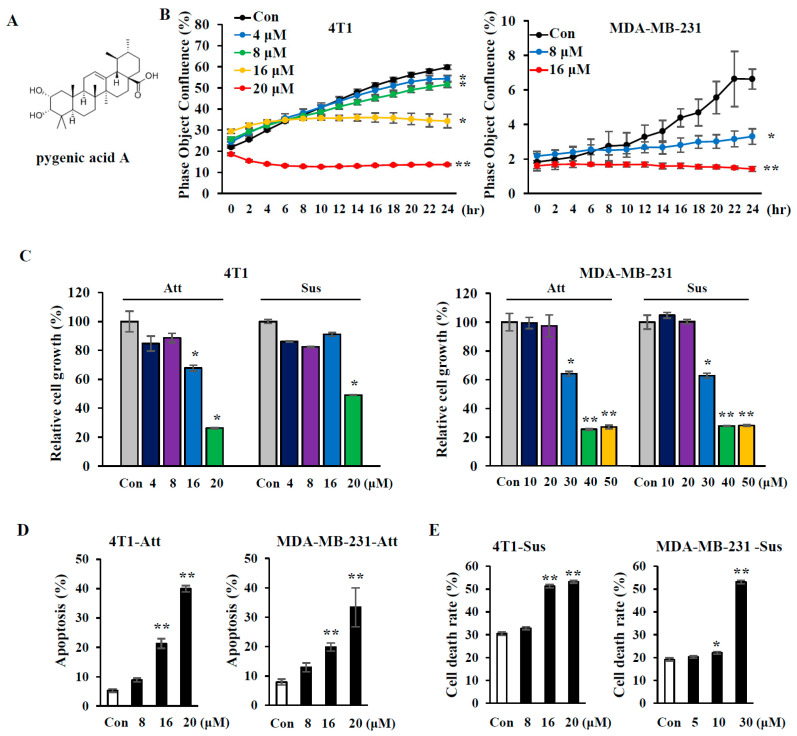

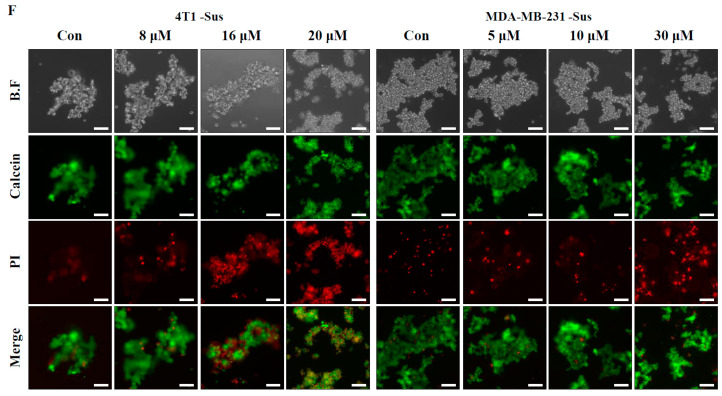
Effects of pygenic acid A (PA) on cell growth and anoikis in metastatic breast cancer cell lines. (**A**) Structure of pygenic acid A (PA) (**B**) 1 × 10^4^ cells/well were seeded for 24 h, and then treated with indicated concentrations (0–20 µM) of PA for 24 h. Cell proliferation was measured with the IncuCyte™ system. (**C**–**E**) Cells in either attachment or in suspension culture were treated with 0–50 µM PA for 24 h. Cells were subjected to either MTS assay for cell growth (**C**), annexin-V/PI staining for apoptosis (**D**), or calcein-AM/PI staining for live/dead cell assay (**E**), as described in the Section 4. (**F**) Cells in suspension culture were treated with 0–30 µM PA for 24 h and stained with calcein-AM/PI, followed by image capture using fluorescence microscopy (green: live cells; red: dead cells). Data are presented as mean values from three independent experiments, and error bars represent standard deviations. Error bars represent standard deviations of the mean of three measurements (* *p* < 0.05, ** *p* < 0.01), Magnification: 100×, Scale bars = 100 µm. Experiments were performed independently three times with comparable results.

Pygenic acid A (PA), also known as 3-epicorosolic acid or corosolic acid (structure shown in Figure 1A) has been used as a traditional oriental medicine for centuries [10]. Although many studies have demonstrated the anti-tumor activity of PA (corosolic acid) [3], there have been few reports of its effects on cancer metastasis. After screening of thousand natural compounds for their ability to sensitize metastatic breast cancer cells to anoikis, we found PA candidates for inducing the apoptosis of cells under suspension conditions (Figure 1). Here, we investigated whether PA has anti-metastatic effects using the metastatic triple negative breast cancer cell lines, MDA-MB-231 and 4T1, which are human and mouse breast cancer cell lines, respectively [11]. First, we evaluated the effect of PA on cell growth using the IncuCyte™ systems. PA treatment decreased the growth of both 4T1 and MDA-MB-231 cells in a dose-dependent manner (Figure 1B). For successful metastasis, cancer cells need to acquire anoikis-resistance so that they can survive in circulation despite being detached from the ECM [12]. Therefore, sensitization of cancer cells to anoikis could be used as a therapeutic strategy to prevent metastasis. To test whether PA could sensitize cells to anoikis, cells in either attachment or suspension cultures were treated with PA, and cell growth was assessed by MTS assay. Cell growth was inhibited in both culture conditions in a dose-dependent manner (Figure 1C). To determine whether this growth inhibition was due to cell death, we performed an apoptosis assay by flowcytometry of annexin-V/PI-stained cells and found that PA treatment increased the annexin-V-positive population in attached cells (Figure 1D). To assess PA-induced cell death in suspension culture, we performed a live/dead assay by staining cells with calcein-AM/PI (Figure 1E,F), and live cells and dead cells were distinguished by green (calcein-AM) and red colors (PI), respectively. PA treatment resulted in an increase in PI-positive cells, indicating enhanced anoikis in suspended cells (Figure 1E). Cancer cells form cell aggregates, a critical process for anoikis-resistance, when they detach from ECM [9]. As shown in Figure 1F, PA treatment inhibited cell aggregate formation, and thus increased the number of dead cells, as shown by staining with PI (red color), indicating that PA sensitizes both 4T1 and MDA-MB-231 cells to anoikis. All these data indicate that PA not only induces apoptosis in the attached condition but also sensitizes cells to anoikis in the suspended conditions.

### 2.2. PA Decreases Survival-Related Proteins and Increases Caspase 3 Cleavage

**Figure 2:**
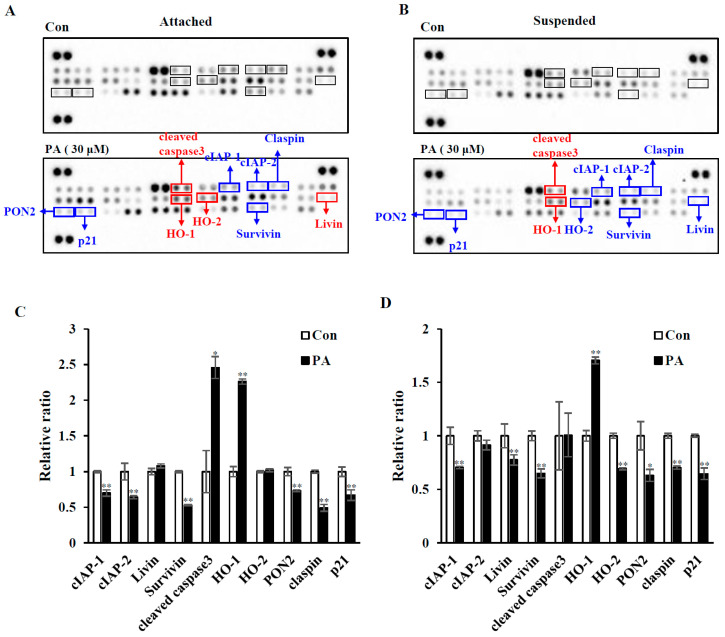
Analysis of apoptosis protein array of PA-treated MDA-MB-231 cells (**A**–**D**) MDA-MB-231 cells in attached (**A**) or in suspended culture (**B**) were treated without (control) or with 30 µM PA, and then equal amounts of cellular proteins were subjected to a protein array using the Proteome Profiler Human Apoptosis Array Kit (R&D system), as described in Section 4. Representative scanned images are shown (**A**,**B**). Scanned images of A and B were quantified with a densitometer and expressions relative to the control are shown in (**C**,**D**), respectively. Similar results were observed in three independent experiments. Error bars represent standard deviations of the means of three measurements (* *p* < 0.05, ** *p* < 0.01).

To examine which death pathways are involved in PA-induced apoptosis and anoikis, we first performed an apoptosis-related protein array using the Proteome Profiler Human Apoptosis Array Kit (R&D system). MDA-MB-231 cells either in attached (Figure 2A) or suspended condition (Figure 2B) were treated with 30 µM PA and subjected to protein array. The up-regulated and down-regulated proteins are labeled with red and blue, respectively. The intensity of the arrays was quantified by densitometric analysis, as shown in Figure 2C,D. PA treatment induced the activation of apoptosis pathways regardless of the cell adhesion condition with little difference in signaling. PA decreased the level of cell survival proteins, such as inhibitor of apoptosis family proteins, cIAP1 and cIAP2, survivin, livin, and claspin. PA induced caspase 3 activation as determined by its cleavage. Heme oxygenase-1 and -2 (HO-1 and HO-2) are known to function in cell adaptation to cellular stress, such as oxidative stress, cell detachment from the ECM [13], and ER stress [14]. Unexpectedly, PA upregulated HO-1 in both attached and suspended cells, whereas it downregulated HO-2 in suspended cells. Paraoxonase-2 (PON-2), an anti-oxidative protein [15], is known to protect cells from anoikis [16], it was downregulated by PA treatment in both conditions. However, PA decreased the level of p21, a cell cycle inhibitor. These data indicate that PA downregulates anti-apoptotic proteins and thus activates caspase 3, which leads to cell death in both attached and suspended cells.

**Figure 3:**
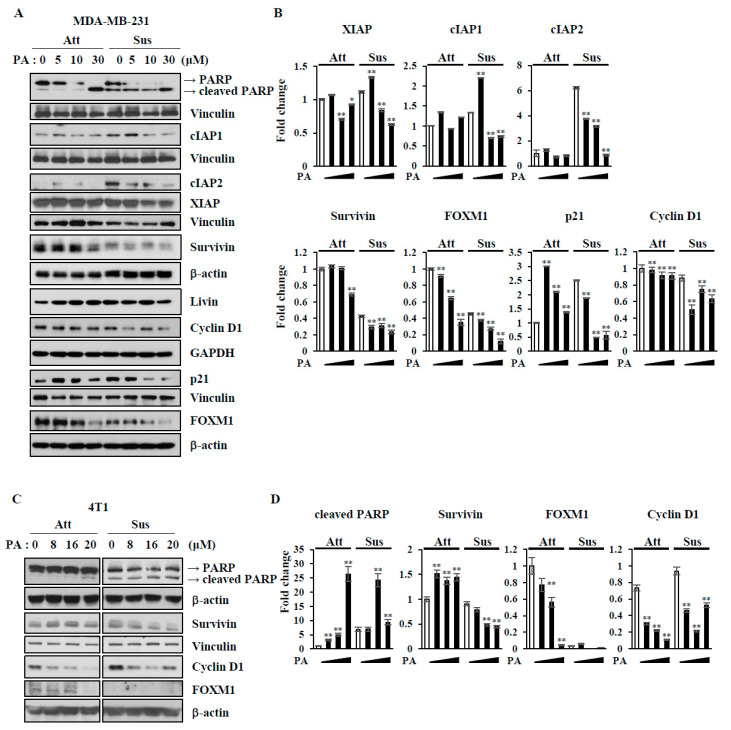
Effects of PA on the expression levels of proteins for cell survival and apoptosis. (**A**,**C**) MDA-MB-231 cells (**A**) and 4T1 cells (**C**) were treated with the indicated concentrations (0–30 µM) of PA for 24 h, and cell lysates were subjected to immunoblotting analysis using the indicated antibodies. (**B**,**D**) The levels of proteins were quantified by densitometry and normalized to reference proteins (actin or vinculin or GAPDH). Error bars represent standard deviations of the mean of three measurements (* *p* < 0.05, ** *p* < 0.01). Similar results were observed in three independent experiments.

Next, we validated the results from the protein array by immunoblotting analysis (Figure 3). Consistent with apoptosis array data, PA treatment increased PARP cleavage and decreased cIAP family proteins, XIAP, cIAP1, cIAP2, livin, and survivin in both attached and suspended MDA-MB-231 cells (Figure 3A,B). Cyclin D1 and p21, cell cycle check point proteins, have been reported to prevent anoikis [17,18]. Interestingly, p21 was upregulated when cells were cultured in suspension, and it was downregulated by PA. In addition, PA downregulated cyclin D1 in suspended cells, but not attached cells. The forkhead box M1 (FOXM1) is an oncogenic transcription factor, that upregulates genes involved in survival and the cell cycle, such as survivin and cyclin D [19]. Consistent with the results showing that PA decreased levels of both survivin and cyclin D1, PA also decreased FOXM1 in both attached and suspended condition (Figure 3A,B). Similar molecular changes induced by PA were also observed in 4T1 cells, as shown in Figure 3C,D.

### 2.3. PA Decreases p-STAT3, p-Akt, and p-p38 in Suspended Cells

**Figure 4:**
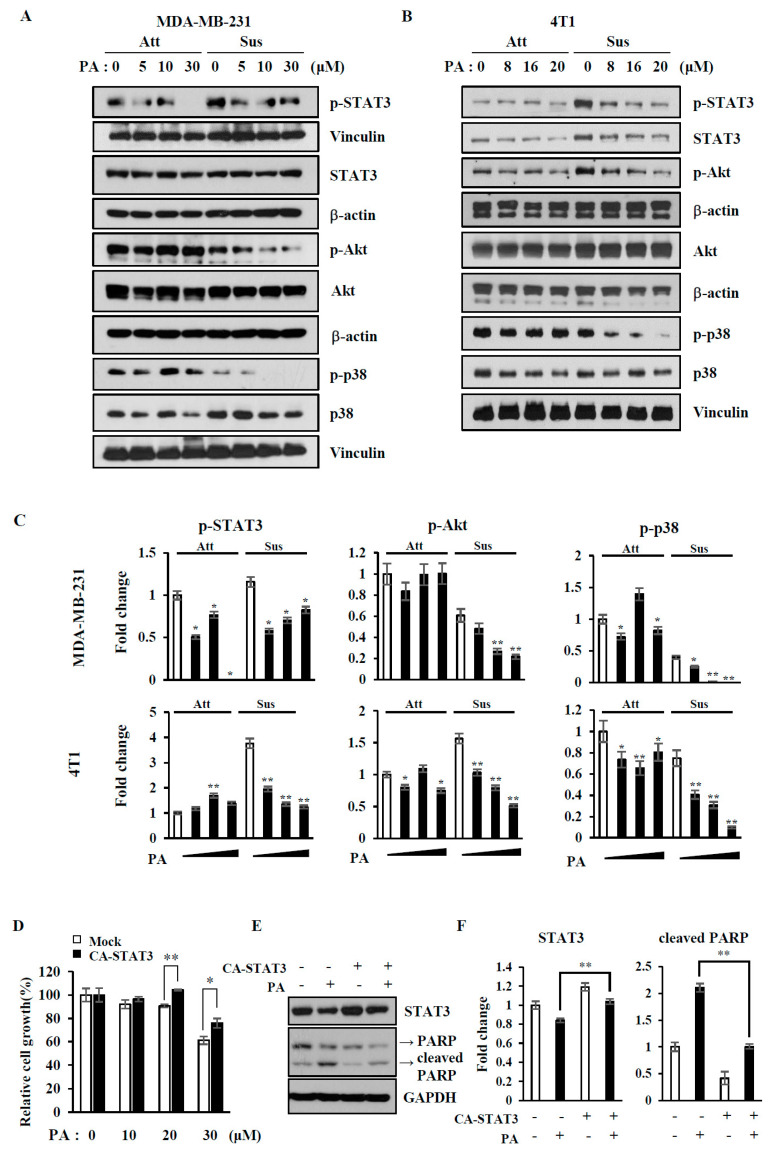
Effects of PA on the activity of STAT3, Akt, and p38 and anoikis resistance, as shown by constitutively active-STAT3 expression (**A**,**B**) MDA-MB-231 cells (**A**) and 4T1 cells (**B**) were treated with indicated concentrations (0–30 μM) of PA for 24 h, and cell lysates were subjected to immunoblotting analysis using the indicated antibodies. The levels of proteins were quantified by densitometry and normalized to a reference protein (**C**). (**D**–**F**) MDA-MB-231 cells were transfected with either vector only (Mock) or constitutively active STAT3 (CA-STAT3) for 24 h. Cells were then suspended and treated with the indicated concentrations of PA for 24 h, followed by MTS assay (**D**) or immunoblotting analysis using the indicated antibodies (**E**) and quantification of levels of proteins by densitometry (**F**). CA-STAT3 and/or PA treatment were marked with “+”. Similar results were observed in three independent experiments. Error bars represent standard deviations of the mean of three measurements (* *p* < 0.05, ** *p* < 0.01).

Because the activation of several signaling pathways, including Akt, STAT3, and p38, plays a role in anoikis resistance, we examined changes in their activation following PA treatment under either attached or suspended conditions (Figure 4A–C). The level of p-STAT3 was enhanced upon cell detachment in both MDA-MB-231 and 4T1 cells and reduced by PA treatment. Although PA reduced the levels of both p-Akt and p-p38, the degree of inhibition was much greater in the suspended cells than in the attached cells. Because p-STAT3 levels increased upon cell detachment and PA decreased p-STAT3, we tested whether the ectopic expression of constitutively active-STAT3 (CA-STAT3) could attenuate anoikis in MDA-MB-231 cells. PA-induced cell death was reduced upon active STAT3 expression with a decrease in PARP cleavage (Figure 4D–F). These data suggest that PA induces cell death/anoikis via downregulation of anti-apoptotic proteins and inactivation of survival-related proteins.

### 2.4. ER-Stress and Autophagy Are Associated with PA-Induced Cell Death

**Figure 5:**
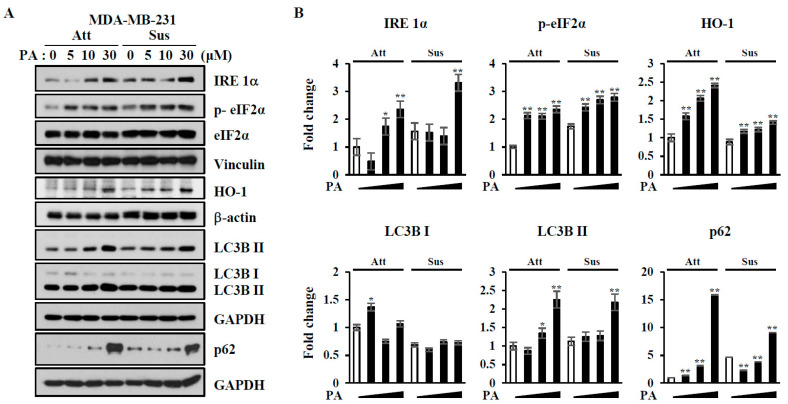

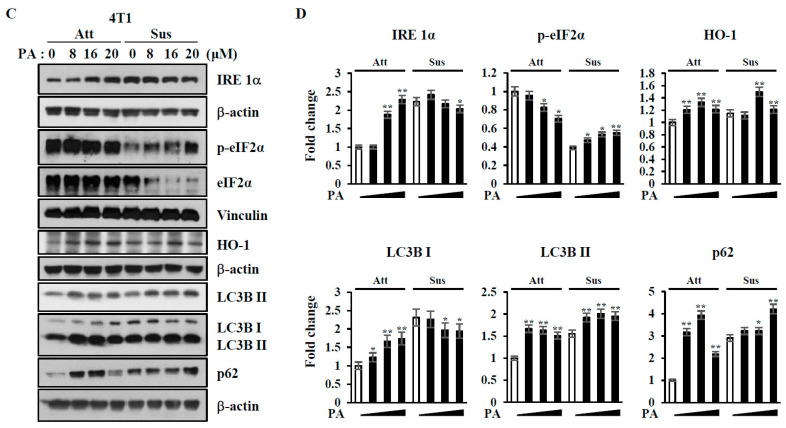
Effects of PA on the expression of markers of ER-stress and autophagy. (**A**,**C**) MDA-MB-231 cells (**A**) and 4T1 cells (**C**) were treated with the indicated concentrations (0–30 µM) of PA for 24 h, and cell lysates were subjected to immunoblotting analysis using the indicated antibodies. (**B**,**D**) The levels of proteins were quantified by densitometry and normalized to reference proteins (actin or vinculin). Error bars represent standard deviations of the mean of three measurements (* *p* < 0.05, ** *p* < 0.01). Similar results were observed in three independent experiments.

Cell detachment from the ECM is considered to be a type of cell stress, and cells have adapted to this stress by activating the integrated stress responses, such as by decreasing protein synthesis, caused by phosphorylation of eIF2α [20]. PON2 is cytoprotective against ER-stress-induced apoptosis and anoikis [16], and its expression was reduced by PA treatment (Figure 2). In addition, corosolic acid is known to induce ER stress [21] one of the important mechanisms of apoptosis, which triggers autophagy [22]. Therefore, it is possible that ER stress and autophagic cell death might be activated to sensitize cells to anoikis. To investigate the effects of PA on the ER stress response and autophagy in MDA-MB-231 and 4T1 cells, we examined the levels of ER stress response proteins, such as IRE1α and p-elF2α, and autophagy markers, including LC3B I, LC3B II, and p62 (Figure 5A–D). PA increased the concentrations of all these proteins in attached MDA-MB-231 and 4T1 cells, although some proteins were regulated differently in the suspended cells. Protein levels of IRE-1α increased upon cell detachment and further increased following PA treatment in MDA-MB-231 cells. PA also increased levels of p-elF2α in suspended cells of both lines. HO-1, LC3B II, and p62 were upregulated by PA in both cell lines, regardless of cell attachment. These results suggest that PA treatment induces ER stress and autophagy not only in attached but also in suspended cells.

### 2.5. PA Inhibits Various Metastatic Characteristics and Tumor Metastasis in Syngeneic Mouse Model

**Figure 6:**
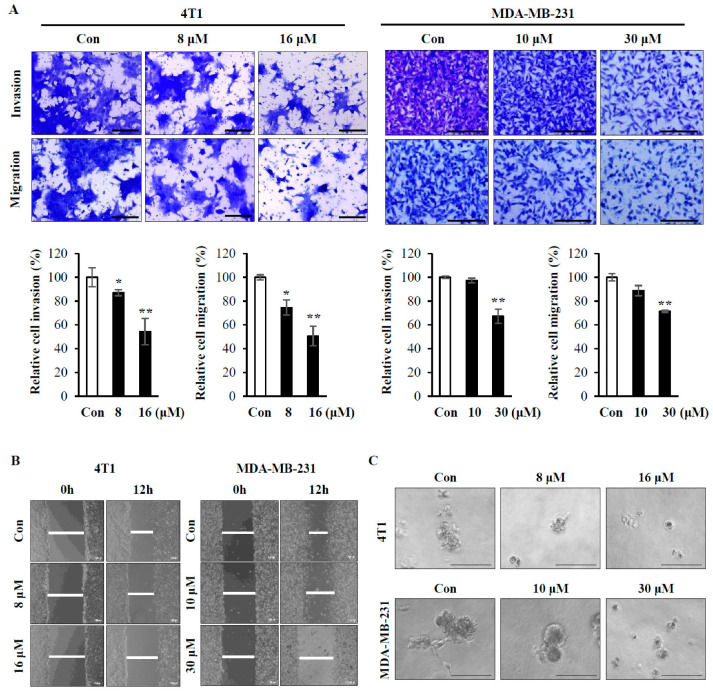
Effects of PA on invasion, migration, wound healing, and growth in 3D culture conditions (**A**,**B**) 4T1 cells and MDA-MB-231 cells (4 × 10^4^ cells/well) were plated into the Transwell upper chamber with the indicated concentrations of PA, followed by incubation for 20 h. Invaded and migrated cells were then stained with 0.5% crystal violet. For invasion assays, the upper chambers were precoated with matrigel. Representative images were taken and scanned to quantify three different areas (**A**). Scale bars = 50 µm. (**B**) Confluent cells were grown in a 12 well plate, and cell monolayers were scratched with a pipet tip. Cells were then incubated with or without PA for 12 h, followed by image capture. Magnification = 50×, Scale bar = 100 µm. (**C**) 2 × 10^3^ cells in 300 μL of 5% matrigel were plated in the matrigel-coated 8-well chamber with the indicated concentration of PA, as described in Section 4. Colony images were captured after 6 days of incubation. Scale bar = 100 µm. Similar results were observed in three independent experiments. Error bars represent standard deviations of the mean of three measurements (* *p* < 0.05, ** *p* < 0.01).

**Figure 7:**
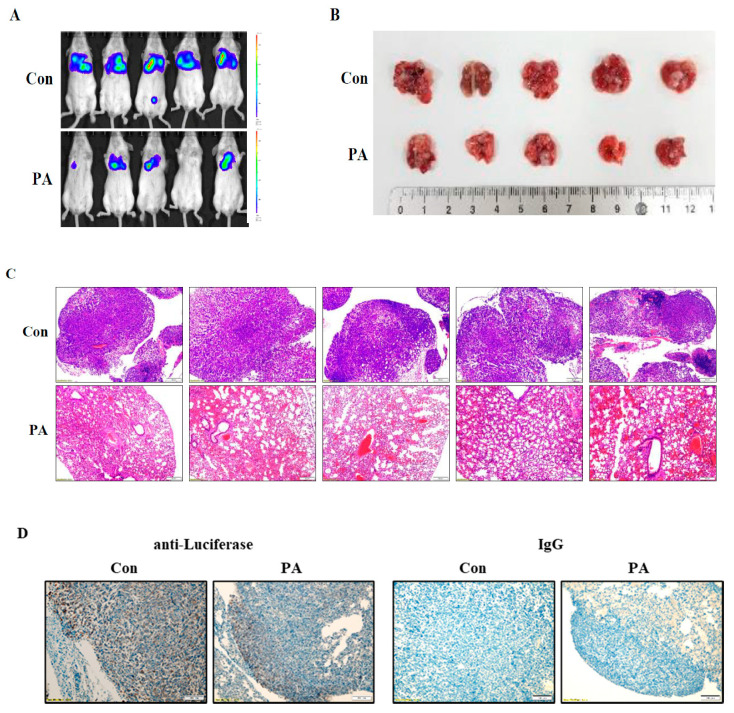
Effects of PA on lung metastasis in a syngeneic mouse model. (**A**) Luciferase-labeled 4T1 cells were pretreated with PA for 4 h and injected into the tail vein of syngeneic Balb/c mice (*n* = 10). Bioluminescence images of luciferase expression were captured. (**B**) All mice were sacrificed after 4 weeks, and lungs were extracted. (**C**) Lung tissues were processed for immunohistochemistry of H&E staining. Scale bars = 200 μm. (**D**) Tumor tissues were fixed and stained either with anti-luciferase antibody or with nonspecific isotype IgG as a control. Scale bars = 200 μm. Similar results were observed in two independent experiments. Similar results were observed in two independent experiments.

Tumor metastasis is a complex process which involves cell migration, invasion, and detachment from the ECM and re-adhesion followed by proliferation at secondary sites [23]. To test whether PA inhibits these metastatic features, we performed cell invasion, migration, and wound healing assays using breast metastatic cancer cell lines in the absence or presence of PA. PA treatment significantly decreased cell invasion and migration (Figure 6A), and wound healing ability was also reduced by PA treatment in a dose-dependent manner in both MDA-MB-231 cell and 4T1 cells when compared with the control (Figure 6B). We further examined whether PA could affect cancer cell growth in 3D culture, which more closely resembles in vivo cell environments, and its use in drug discovery is thereby expected to facilitate the discovery of candidates that are more likely to be successful in therapy [24]. PA treatment resulted in the dose-dependent decrease in the sizes of colonies of both MDA-MB-231 and 4T1 cells. In addition, control MDA-MD-231 cells formed a highly invasive phenotype (star-like colony), which was diminished by PA treatment (round colony) (Figure 6C). To examine the anti-metastatic effects of PA in vivo, we injected PA-treated 4T1 cells into the tail veins of mice and examined the occurrence of pulmonary metastasis of control versus PA-treated cells. It is of interest that the degree of lung metastasis of luciferase-labeled 4T1 cells decreased following PA treatment when compared with the control group (Figure 7A). Lung metastasis, as shown in the lung images, was also reduced by PA (Figure 7B). Consistent with these observations, there was reduced tumor metastasis as determined by H&E-stained lung histology (Figure 7C). Because we injected 4T1 cells expressing luciferase, we stained lung tissues with anti-luciferase antibodies to verify the tumor mass made by 4T1 cells, as shown in Figure 7D. tumor nodules were positive for luciferase in both control and PA-treated tissue. All these data indicate that PA inhibits the metastatic potential of cells by sensitizing them to anoikis and attenuating cell invasion, migration, and growth in 3D culture, thus reducing tumor metastasis.

## 3. Discussion

Tumor metastasis is a major cause of death in cancer patients and a barrier to discovering a cure for cancer [25]. Therefore, the inhibition of metastasis is important not only for the control of cancer recurrence but also as a cancer treatment. Anoikis is a form of apoptosis caused by cell detachment from the ECM. Normal cells are sensitive to anoikis when they are detached from the ECM, which is important for normal development. However, some cancer cells become resistant to anoikis and survive even without ECM attachment. The acquisition of anoikis resistance is a prerequisite for cancer cells, especially for circulating tumor cells because they must survive during circulation to successfully metastasize [7]. In this regard, the sensitization of cancer cells to anoikis could prevent metastasis. By screening 1000 compounds from plant extracts, we first demonstrated that PA effectively sensitizes metastatic breast cancer cells to anoikis and inhibits lung metastasis in the syngeneic mouse model.

Many studies have demonstrated that PA has antitumor and anti-proliferative effects in many cancer cells, such as colon, leukemia, and osteosarcoma cells [4,21,26]. PA is known to exert its antitumor effects via STAT3 inactivation, HER2 downregulation, and an increase in intracellular reactive oxygen species (ROS) levels [27]. In these studies, the effects of PA on cell death and growth inhibition were examined using attached cells. In our study, we demonstrated the anti-metastatic effects of PA using suspended cells in addition to attached cells. As shown in Figure 1, PA induced cell death in both attachment and suspension culture conditions, indicating that PA sensitizes metastatic breast cancer cells to anoikis. We also tested whether PA is toxic to the MCF-10A cell line, which is a human normal breast epithelial cell line. As shown in Supplementary Figure S1, PA treatment (0–40 μM) did not affect cell viability, indicating little toxicity in the normal cells. In addition, Ma et al. demonstrated that corosolic acid does not induce mouse toxicity and organ damage [21].

Anoikis occurs either via mitochondrial pathways or death receptor pathways [28]. Anoikis resistance involves various signaling pathways and results in the inhibition of mitochondrial pathways via the upregulation of anti-apoptotic proteins and downregulation of pro-apoptotic proteins. Our apoptosis protein array revealed that PA treatment decreased mostly anti-apoptotic proteins, such as cIAPs, claspin, p21, PON-2, and survivin, regardless of cell attachment (Figure 2). We validated some of these array data by immunoblotting and found that PA decreased levels of cIAP1, cIAP2, and survivin, and thus increased PARP cleavage in both attached and suspended cells (Figure 3). Corosolic acid induces apoptosis through the mitochondrial pathway in cervical cancer and hepatocarcinoma cells [29], and we also observed that PA decreased mitochondrial membrane potentials in MDA-MB-231 cells (Supplementary Figure S2). Additionally, we found that the levels of survivin and cyclin D1 decreased and could be regulated by both STAT3 and FOXM1. Interestingly, PA inactivated STAT3 and decreased FOXM1 levels (Figure 3 and Figure 4). It is very intriguing that PA downregulates FOXM1 because it is an oncogenic transcription factor that regulates the expression of cell cycle-related genes and survival genes, such as cyclins and survivin [30]. Although FOXM1 was downregulated upon cell detachment, PA further decreased FOXM1 levels. It is also interesting that PA decreased p21 and cyclin D1 levels, because p21 and cyclin D1 are known to prevent anoikis [31]. Because p21 and cyclin D1 are linked to cell cycle regulation, we examined the effects of PA on the cell cycle. For attached cells, PA treatment increased the number of cells in sub G1 and Go/G1, while S and G2/M1 cells were decreased (Supplementary Figure S3), indicating that PA inhibits the cell cycle and activates apoptosis. In glioma cells, PA decreased p-STAT3 in the attached cells [32], and we also observed that PA decreased STAT3 activation in the attached MDA-MB-231 cells (Figure 4), which was attenuated by the expression of active STAT3 along with an increase in survivin (Supplementary Figure S4). STAT3 activation is important for anoikis resistance [33], and cell detachment activated STAT3, which was inactivated by PA (Figure 4). Ectopic expression of constitutively active STAT3 attenuated PA-induced anoikis sensitivity with decreased caspase 3 activation, as determined by PARP cleavage (Figure 4). These data indicate that PA-induced STAT3 inactivation is associated with anoikis sensitization.

Reactive oxygen species (ROS) are important for anoikis resistance [34]. Because NOX4 upregulation has been linked to anoikis resistance, we tested whether PA affects NOX family members. As shown in Supplementary Figure S5, although NOX4 was upregulated by cell detachment, PA did not inhibit NOX4 upregulation and, rather, it decreased NOX2 levels regardless of adhesion. Cell detachment induced genes involved in the stress response, such as p-elF2α, IRE1α, and HO-1 (Figure 5). HO-1 upregulation is known to prevent cells from anoikis and promote the metastasis of human fibrosarcoma HT1080 cells via ameliorating oxidative stress [13]. Consistent with this previous report [13], we observed that HO-1 increased slightly upon cell detachment (Figure 5). However, PA did not attenuate this increase in HO-1 and, rather, it further increased the concentration of HO-1 in MDA-MB-231 and 4T1 cells, regardless of cell adhesion. Although HO-1 is known to play a cytoprotective role and thereby regulate proliferation and metastasis [13], it has also been reported that HO-1inhibits breast cancer cell proliferation through the inhibition of indoleamine 2,3-deoxygenase [35]. Therefore, it is possible that PA-induced HO-1 might function in the induction of cell death. Instead, PA treatment decreased other antioxidative proteins, HO-2 and PON-2 (Figure 2), and their roles in PA-induced cell death remain to be further elucidated.

PON-2, an antioxidant protein, has a protective role in ER stress from apoptosis [36], and overexpression of PON-2 inhibits tumor development by inhibiting insulin-like growth factor-1 (IGF-1) expression and signaling in ovarian cancer cells [37]. In addition, corosolic acid is known to induce ER stress [21]. In our study, PA treatment decreased the concentration of PON-2 in both attached and suspended cells (Figure 2). Upon cell detachment, both ER stress and autophagy occur, and these events are important for anoikis resistance [38]. ECM detachment is known to activate the ER stress kinase PERK, which protect cells from anoikis by activating detachment-induced autophagy [38]. PERK, a kinase for elF2α, increases p-elF2α levels, which results in a global decrease in protein translation but an increase in the transcription factor ATF4, which induces autophagy regulatory proteins and HO-1 [15]. In our study, cell detachment appeared to activate ER stress and autophagy, as determined by increases in their markers, such as p-elF2α and IRE 1α (ER stress) and LC3B II (autophagy) (Figure 5). PA treatment further upregulated these proteins in both attached and suspended MDA-MB-231 cells (Figure 5). PA increased IRE 1α and p-elF2α levels in the attached and suspended 4T1 cells, respectively. These data indicate that ER stress and autophagy are associated with PA-induced cell death in both attached and suspended cells. It is interesting that p62, a well-known autophagy substrate, undergoes crosstalk with both autophagy and apoptosis [39]. Unsuccessful autophagy flux, such as defects in the fusion of an autophagosome with a lysosome, leads to p62 accumulation, thereby causing a switch from autophagy to synergistic induction of apoptosis [39,40]. PA increased p62, regardless of cell attachment (Figure 5), and it is possible that PA-induced p62 accumulation might switch autophagy to the induction of apoptosis.

Our data also showed that PA inhibits cell invasion and migration, which are important processes for metastasis (Figure 6). Furthermore, PA attenuated lung metastasis of 4T1 cells when injected via the tail vein of a syngeneic mouse (Figure 7), indicating inhibitory effects of PA not only on anoikis resistance but also on the ability of cancer cells to penetrate blood vessels and be colonized for growth. In conclusion, our data suggest that PA can act as an effective apoptotic compound in human and mouse metastatic breast cancer cells through the inhibition of Akt, STAT3, and p38 MAPK and activation of the ER stress response. Although, further studies are needed to fully elucidate the molecular mechanism underlying anoikis sensitization, our study suggests that sensitization of cancer cells to anoikis is a useful strategy to prevent tumor metastasis and that PA is one of the candidates for the control of metastasis.

## 4. Materials and Methods

Materials Pygenic acid A was gifted from NIKOM (National Institute for Korean Medicine Development, 94 Hwarang-ro, Gyeongsan-si, Gyeongsangbuk-do, Korea). Anti-LC3B, anti-p62, anti-PARP, anti-survivin, anti-Livin, anti-XIAP, anti-cIAP1, anti-cIAP2, anti-p21, anti-cyclin D1, anti-FOXM1, anti-phospho STAT3, anti-STAT3, anti-phospho Akt, anti-Akt, anti-phospho p38, anti-p38, anti-phospho eIF2α, anti-eIF2α, anti-IRE1α, anti-GAPDH antibodies were purchased from Cell Signaling Technology (Beverly, MA, USA). Anti-HO-1 antibody, Horseradish peroxidase (HRP)-conjugated rabbit IgG, and HRP-conjugated mouse IgG were purchased from Enzo Life Sciences (Farmingdale, NY, USA). Anti-Vinculin and firefly luciferase, Rabbit IgG, polyclonal-Isotype Control antibodies were purchased from Abcam (Cambridge, UK). Anti-β-actin antibody, Dimethyl sulfoxide (DMSO), and Poly (2-hydroxyethyl methacrylate) (Poly-HEMA) were purchased from Sigma-Aldrich Corporation (St. Louis, MO, USA).

### 4.1. Extraction and Isolation of Pygenic Acid A

The ^1^H and ^13^C NMR spectra were recorded on a Jeol ECA-500 MHz NMR instrument, operating at 500 MHz for ^1^H NMR and 125 MHz for ^13^C NMR (Jeol, Tokyo, Japan) with tetramethylsilane (TMS) as internal standard. Electron ionization-mass spectrometry (EI-MS) was measured using a Waters Q-TOF micro mass spectrometer. Silica gel (Kieselgel 60, 230-400 mesh, Merck, Germany) were used for column chromatography. High performance liquid chromatography (HPLC) was performed with Agilent 1260 series (Agilent Technologies, CA, USA). The *Prunella vulgaris* were purchased from a commercial herbal market Humanherb, Gyeongsan in 2013. The aerial part of *P. vulgaris* (300 g) were extracted two times with 95% methanol (2 L) at room temperature for 3 h. After filtration, the crude methanol (MeOH) extracts were concentrated under rotary evaporator and vacuum drier at 40 °C. The crude MeOH extracts (22.4 g) were suspended in distilled water (500 mL) and solvent partitioned with same volume of dichloromethane (CH2Cl2). The CH_2_C_l2_ soluble fraction (8.4 g) was separated into four fractions (PVC 1–4) by chromatography on a silica gel column (Kieselgel 60, Merck) with ethylacetate (EA) and methanol (MeOH) solvent (EA:MeOH = 10:0 to 0:10). The PVC 2 (2.5 g) was further separated by preparative reversed-phase high performance liquid chromatography (YMC ODS column 250 mm × 10 mm id., 50 μm; flow rate, 3 mL/min; mobile phase: MeOH:H_2_O = 7:3; UV detection at 254 nm) to yield the active compound (60 mg). The active compound was identified as a pygenic acid A by comparing its various spectroscopic techniques, including ^1^H and ^13^C NMR and EI-MS with the before literature [41].

### 4.2. Cell Culture

MDA-MB-231 was gifted from Dr. Hyunchul Jang (National Cancer Center, Gyeonggi-do, Korea). 4T1-luc was kindly gifted from Dr. Byungheon Lee (Kyungpook National University, Daegu, Korea). MDA-MB-231 cells and 4T1-luc cells were grown in RPMI-1640 (Hyclone, Logan, UT, USA) supplemented with 10% FBS (Hyclone, Logan, UT, USA), 100 units/mL penicillin, 100 μg/mL streptomycin, and 0.25 μg/mL amphotericin B (Antibiotics-Antimycotic, Gibco Laboratories Co., Grand Island, NY, USA) at 37 °C in a humidified atmosphere containing 5% CO_2_.

### 4.3. Suspension Culture

Suspended cell culture were obtained and modified from previous study [8]. Tissue culture plates (60 mm) were coated with 400 μL of poly-HEMA (50 mg/mL in 95% ethanol) and dried for overnight in a laminar flow at room temperature (RT). Cells were trypsinized into a single cell suspension and 4 × 10^5^ cells were plated on poly-HEMA-coated dishes. After 24 h, cells were harvested by centrifugation and processed for cell viability, flow cytometric analysis, and protein analysis.

### 4.4. Cell Viability and Proliferation Assays

For the cell viability assay, cells were plated in a 96-well culture plate for 24 h before treatment (approximately 70% confluence). The cells were exposed to indicated concentration of PA in RPMI 1640 medium containing 1% FBS for 24 h. For the cell proliferation assay, the cells were determined using Incucyte™. Cell growth was determined using the CellTiter 96 Kit (MTS, 3-(4,5-dimethylthiazol-2-yl)-5-(3-carboxyme-thoxyphenyl)-2-(4-sulfophenyl)-2H-tetrazolium; Promega, Madison, WI, USA) as previously described.

### 4.5. Anoikis Assay

Cells were exposed to indicated concentration of PA in a 96-well low-attached culture plate. After 24 h, cell growth was determined using MTS as previously described.

### 4.6. Live/Dead Viability Assay

Cells were plated on 24-well low-attached plates in suspended (2.5 × 10^4^ cells/0.5 mL/well). After 12 h, live or dead cells were analyzed by Live/Dead viability assay. Briefly, cells were stained with 1 μM PI and 1 μM Calcein AM for 30 min at room temperature. The labeled cells with two-color fluorescence (green-live cells, red-dead cells) were analyzed by an Axio Observer Z1 fluorescence microscope (Carl Zeiss Microimaging, Thornwood, NY, USA) and an Axion Vision camera (Axion Technologies, Houston, TX, USA). Cells counted using Arthur Novel fluorescence cell counter (NanoEnTek, Seoul, Korea).

### 4.7. Apoptosis Assay

Treated cells were harvested and incubated for 15 min at RT with PI (5 µg/mL) and fluorescein isothiocyanate (FITC)-conjugated annexin-V reagent (2.5 µg/mL) in binding buffer followed by flow cytometry analysis. The data were analyzed with Cell Quest Software (BD Bioscience, San Jose, CA, USA).

### 4.8. Human Apoptosis Proteome Profiler Array

We determined apoptosis-related proteins using the Proteome Profiler Human Array (R&D Systems, Minneapolis, MN, USA). After treated, cell lysates (200 μg) were extracted and applied per array set comprised of two nitrocellulose membranes with spotted capture antibodies. The pixel density of spots was quantified using Image J. Spot densities were normalized against respective reference array spots and then against control.

### 4.9. Transfection of Cells

Constitutively active STAT3 (CA-STAT3)/pcDNA3 construct was a kind gift from Dr. Sang Kyu Ye (Seoul National University College of Medicine, Seoul, Korea) and was described previously. MDA-MB-231 cells were transfected with CA-STAT3/pcDNA3 or mock vector plasmids using Lipofectamine 3000 reagent (Invitrogen, Carlsbad, CA, USA) for 24 h.

### 4.10. Immunoblot Analysis

After washing with ice-cold PBS, cells were lysed with 1x SDS lysis buffer (10 mM Tris, 1 mM EDTA, 0.5 mM Na_3_VO_4_, 1 mM DTT, 1% SDS, 10% glycerol) and boiled for 5 min. Protein concentration of each sample was determined by micro BCA protein assay reagent (Thermo scientific, Rockford, IL, USA). 20 μg of total cellular protein was separated by 8 or 12% SDS-PAGE and transferred to PVDF membrane (Millipore, Bedford, MA, USA). The membranes were blocked for 60 min at RT in tris-buffered saline and tween 20 (TBST) containing 5% non-fat dried milk. The membranes were incubated with the primary antibody for overnight at 4 °C, washed three times with TBST for 10 min, incubated with HRP-conjugated goat anti-mouse IgG or goat anti-rabbit IgG secondary antibodies for 1 h at RT, and then washed with TBST four times for 10 min. The labeled proteins were visualized by the enhanced chemi-luminescence method.

### 4.11. Transwell Migration and Invasion Assays

Transwell assay was employed (8 μM pore, Corning, Corning, NY, USA). For migration assays, 4T1 cells or MDA-MB-231 cells were pre-treated and resuspended in 100 μL 1% serum RPMI medium containing control or indicated PA. Each sample contained 4 × 10^4^ cells, which were cultured in the Transwell upper chamber. The lower chamber was filled with RPMI 1640 supplemented with 10% FBS. Following 20 h incubation, the lower chamber was isolated, and cells were fixed using 10% formaldehyde at room temperature (RT) for 10 min. Cells were then stained with 0.5% crystal violet. Results represented the mean of area in three different areas under light microscope (magnification, ×25.2). For invasion assays, the chambers were pre-coated with 80 μL of matrigel (1:10 dilution in serum-free RPMI medium). Protocols were similar as described in the migration assay.

### 4.12. Wound Healing

4T1 cells or MDA-MB-231 cells were seeded in 6-well plates and cultured to 80–90% confluence. Cells were pre-treated with either control (DMSO) or PA for 8 h. The cellular layer in each plate were scratched using plastic pipette tip. After re-treated, the migration of the cells at the edge of the scratch was analyzed at 12 h. The images of the cells were captured using phase contrast microscopy.

### 4.13. 3D Culture Assay

4T1 cells or MDA-MB-231 cells were treated with control or PA and 3D culture assays were performed in 8 well chamber slides (Nunc™ Lab-Tek™, Thermo Fisher Scientific, Waltham, MA, USA) by placing 2 × 10^3^ cells in 300 μL of 5% matrigel onto a base layer 300 μL of 100% matrigel. The plates were then incubated in a 5% CO_2_ atmosphere at 37 °C for 6 days. Images were taken by using an Axio Observer Z1 fluorescence microscope (Carl Zeiss Microimaging, Thornwood, NY, USA) and an Axion Vision camera (Axion Technologies, Houston, TX, USA).

### 4.14. Lung Metastasis In Vivo

Balb/c mice were intravenously injected with 1 × 10^4^ 4T1-luc cells in 200 μL of PBS via tail vein injection and the cells were treated with control (DMSO) or 16 μM PA. After 18 days, the mice were sacrificed, and lungs were harvested, and fixed 4% paraformaldehyde and stained with hematoxylin-eosin (H&E) staining solution (Sigma, St. Louis, MO, USA). This study was reviewed and approved by the Institutional Animal Care and Use Committee (IACUC) of National Cancer Center Research Institute (NCCRI) and IACUC approval number is NCC-19-181D and IACUC approval date is 30/09/2019. NCCRI is an Association for Assessment and Accreditation of Laboratory Animal Care International (AAALAC International) accredited facility and abides by the Institute of Laboratory Animal Resources (ILAR) guide.

### 4.15. Immunohistochemical Staining

The lungs were fixed with 4% paraformaldehyde for 24 h at 4 °C. For immunostaining, after the antigen retrieval process with citrate buffer (pH 6.0) and endogenous peroxidase blocking with 3% hydrogen peroxide, tissue sections were incubated with 1% BSA blocking solution in PBS (*v*/*v*) for 30 min at room temperature, and then with anti-firefly luciferase antibody (Abcam, 1:5000 in blocking solution) overnight at 4 °C in a humidified chamber. Sections were rinsed three times with a washing buffer (1% BSA, 0.1% cold fish skin gelatin, 0.5% Triton ×-100 in PBS) and then incubated with biotinylated secondary antibody (Vector Laboratories; Burlingame, USA, 1:200 in blocking solution) overnight at 4 °C. An avidin-biotin peroxidase enzyme complex was prepared and applied according to manufacturer’s instructions (Vectastain Elite ABC kit). Finally, sections were incubated for 5 min in a DAB/hydrogen peroxide substrate solution (prepared according to manufacturer’s instructions, Vectastain DAB substrate kit, Vector Laboratories). Sections were mounted in an aqueous mountant (Vectashield, Vector Laboratories) and observed under a microscope (Olympus, Tokyo, Japan).

### 4.16. Statistical Analysis

Comparison between two groups were performed using Student’s *t*-test. Statistical significance was defined as * *p* < 0.05 and ** *p* < 0.01. Data represent average values and standard deviations (error bars) obtained from three independent experiments.

## Supplementary Materials

Supplementary materials can be found at https://www.mdpi.com/1422-0067/21/22/8444/s1.
