# Supplementary material for: Pygenic Acid A (PA) Sensitizes Metastatic Breast Cancer Cells to Anoikis and Inhibits Metastasis In Vivo
Source: Int J Mol Sci. 2020 Nov 10;21(22):8444. doi: 10.3390/ijms21228444 (PMC7696818; doi:10.3390/ijms21228444)
Supplement: Supplementary file 1 [file ijms-21-08444-s001.zip › ijms-987975-supplementary.docx]

**Supplementary Information**

**Cell culture** MDA-MB231 was gifted by Dr. Hyunchul Jang (National Cancer Center, Republic of Korea). 4T1-luc cell was kindly gifted by Bynugheun Lee (Kyungpook National University, Republic of Korea). MCF-10A cell was a kind gift from Aree Moon (Duksung Women’s University, Republic of Korea). MDA-MB-231 cells and 4T1-luc cells were grown in RPMI (Hyclone, Logan, UT) supplemented with 10% FBS (Hyclone, Logan, UT), 100 units/ml penicillin, 100 μg/ml streptomycin, and 0.25 μg/ml amphotericin B (Antibiotics-Antimycotic, Gibco Laboratories Co., Grand Island, NY) at 37°C in a humidified atmosphere containing 5% CO_2_. MCF-10A cells were grown in DMEM/F12 (Invitrogen) supplemented with 5% horse serum (Invitrogen), 1% penicillin/streptomycin (Invitrogen), 0.5 μg/ml hydrocortisone (Sigma), 100 ng/ml cholera toxin (Sigma), 10 μg/ml insulin (Sigma), and 20 ng/ml human EGF (Invitrogen) at 37°C in a humidified atmosphere containing 5% CO_2_.

**Antibodies and Reagents** Pygenic acid A was kindly gifted from NIKOM (National Institute of Korean Medicine Development). Anti-phospho-STAT3, anti-STAT3, anti-Survivin, and anti-GAPDH antibody were purchased from Cell Signaling Technology (Beverly, MA). Anti-NOX2, anti-NOX4, and anti-NOX5 were purchased from Abcam (Cambridge, MA). Horseradish peroxidase (HRP)-conjugated rabbit IgG, and HRP-conjugated mouse IgG were purchased from Enzo Life Sciences (Farmingdale, NY). Anti-Vinculin antibody was purchased from Abcam (Cambridge, UK). Anti-β-actin antibody, Dimethyl sulfoxide (DMSO), and Poly (2-hydroxyethyl methacrylate) (Poly-HEMA) were purchased from Sigma-Aldrich Corporation (St. Louis, MO).

**Suspension culture** Tissue culture plates (60 mm) were coated with 400 μl of poly-HEMA (50 mg/ml in 95 % ethanol) and dried for overnight in a laminar flow at room temperature (RT). Cells were trypsinized into a single cell suspension and 4×10^5^ cells were plated on poly-HEMA-coated dishes. After 24 h, cells were harvested by centrifugation and processed for cell viability, flow cytometric analysis and protein analysis.

**Mitochondrial membrane potential analysis** Cells were trypsinized and suspended in PBS containing 2.5 mM EDTA, 2.5 mM EGTA and 1% BSA. For the measurement of mitochondrial membrane potential changes, cells were incubated with 2µM JC-1 in PBS for 15- 30 min at 37C, followed by flow cytometer analysis (FACSCalibur; Becton Dickinson Bioscience, San Jose, CA). JC-1 is a lipophilic dye and it may be the most sensitive indicator for mitochondrial membrane potential. At low membrane potential, JC-1 occurs as a monomer that emits green fluorescent, and at higher membrane potential JC-1 forms aggregates that emit red fluorescence. The Data were analyzed with Cell Quest Software (BD bioscience, San Jose, CA).

**Cell cycle analysis** Cells were trypsinized and washed with PBS and resuspended with 70% ethanol for fixing. After 30 min cells were stained with RNase free PI (Propidium Iodide) solution and followed by flow cytometer analysis (FACSCalibur; Becton Dickinson Bioscience, San Jose, CA).

**Transfection of cells** Constitutively active STAT3 (CA-STAT3)/pcDNA3 construct was a kind gift from Dr. Sang Kyu Ye (Seoul National University College of Medicine, Seoul, Republic of Korea) and was described previously. MDA-MB-231 cells were transfected with CA-Stat3/pcDNA3 or mock vector plasmids using Lipofectamine 3000 reagent (Invitrogen) for 24 h.

**Cell Viability** Cells were plated on 96-well plates (0.5x10^3^ cells/0.1ml/well). The cells were exposed to indicated concentration of PA in culture media for 24 h. Cell growth was determined using the CellTiter 96 Kit (MTS, 3-(4,5-dimethylthiazol-2-yl)-5-(3-carboxyme-thoxyphenyl)-2-(4-sulfophenyl)-2H-tetrazolium; Promega, Madison, WI, USA) as previously described.

**Immunoblot analysis** After washing with ice-cold PBS, cells were lysed with 1x SDS lysis buffer (10 mM Tris, 1 mM EDTA, 0.5 mM Na3VO4, 1 mM DTT, 1% SDS, 10% glycerol) and boiled for 5 min. Protein concentration of each sample was determined by micro BCA protein assay reagent (Thermoscientific, Rockford, IL). 20 μg of total cellular protein was separated by 8 or 12% SDS-PAGE and transferred to PVDF membrane (Millipore, Bedford, MA). The membranes were blocked for 1 h at RT in tris-buffered saline and tween 20 (TBS-T) containing 5% non-fat dried milk. The membranes were incubated with the primary antibody for overnight at 4℃, washed three times with TBS-T for 10 min, incubated with HRP-conjugated goat anti-mouse IgG or goat anti-rabbit IgG secondary antibodies for 1 h at RT, and then washed with TBS-T four times for 10 min. The labeled proteins were visualized by the enhanced chemi-luminescence method.

**Data analysis** All data points represented the mean value of at least three independent experiments with triplicates for each. Statistical significance was determined by Student’s *t*-test with *p*<0.005.


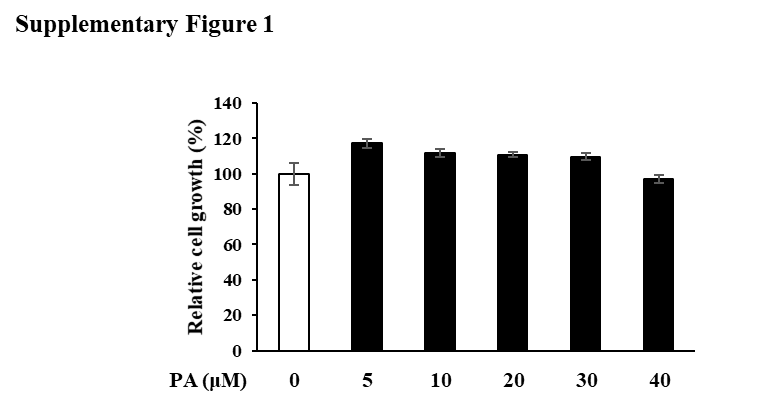


**Supplementary Figure 1. Effects of PA on cell growth of MCF-10A cells** MCF-10A cells were treated without or with indicated concentrations of PA for 24 h and then cells were subjected to MTS assay for cell growth. Data are presented as mean values from three independent experiments, and error bars represent standard deviations. Similar results were observed in three independent experiments.


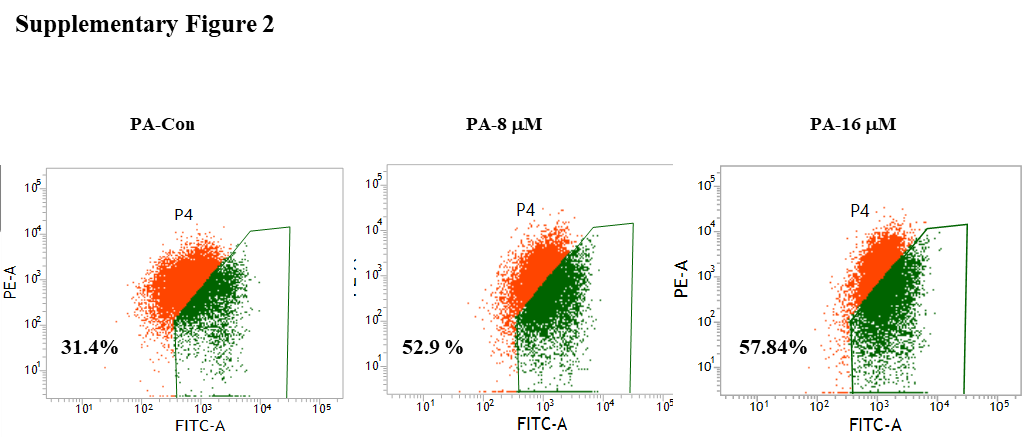


**Supplementary Figure 2. Effects of pygenic acid A (PA) on mitochondrial membrane potential change** 4T1 cells were grown in attached condition and treated with 0-16 μM PA for 24 h. Mitochondrial membrane potential changes (ΔΨ) were measured using the JC-1 kit by flow cytometer. The loss of ΔΨ_m_ was equated with decreased red fluorescence. Similar results were observed in three independent experiments.


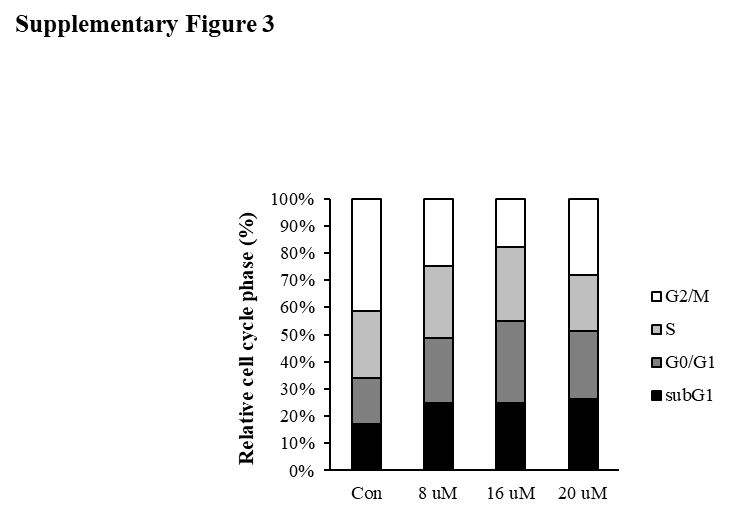


**Supplementary Figure 3. Effects of PA on cell cycle** MDA-MB-231 cells were grown in attached condition and treated with 0-20 μM PA for 24 h, followed by cell cycle analysis using flow cytometry. Similar results were observed in three independent experiments.


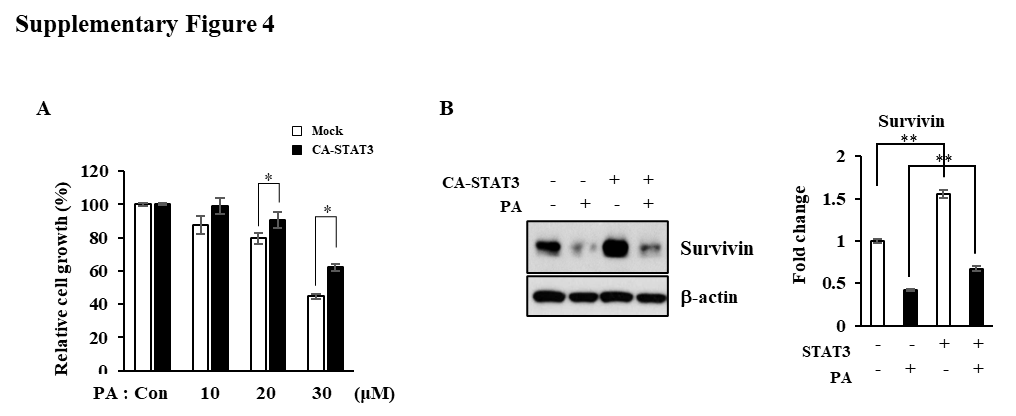


**Supplementary Figure 4. Effects of STAT3 activation on PA-induced cell growth inhibition (A and B)** MDA-MB-231 cells were transfected with either vector only (Mock) or constitutively active STAT3 (CA-STAT3) for 24 h. Cells were then treated with 0-20 μM PA in attached condition for 24 h, followed by MTS assay for cell growth.**(A) or** immunoblotting analysis using the indicated antibodies **(B).** β**-**actin was used as a loading control. The levels of survivin were quantified by densitometry and normalized to β**-**actin. Similar results were observed in three independent experiments. Error bars represent standard deviations of the mean of three measurements (*P<0.05).


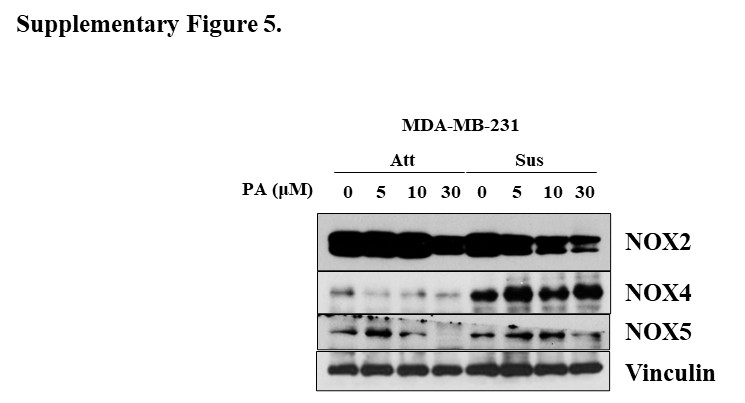


**Supplementary Figure 5. Effects of PA on expression of NOX family proteins** Attached or suspended MDA-MB-231 cells were treated without or with indicated concentrations of PA for 24 h and cell lysates were subjected to immunoblotting analysis using the indicated antibodies. Vinculin was used as a loading control. Similar results were observed in three independent experiments.
